# A Possible Mechanism: Vildagliptin Prevents Aortic Dysfunction through Paraoxonase and Angiopoietin-Like 3

**DOI:** 10.1155/2018/3109251

**Published:** 2018-05-23

**Authors:** Qian Zhang, Xinhua Xiao, Jia Zheng, Ming Li, Miao Yu, Fan Ping, Tong Wang, Xiaojing Wang

**Affiliations:** Key Laboratory of Endocrinology, Ministry of Health, Department of Endocrinology, Peking Union Medical College Hospital, Peking Union Medical College, Chinese Academy of Medical Sciences, Beijing, 100730, China

## Abstract

The collected data have revealed the beneficial effects of dipeptidyl peptidase-4 (DPP-4) inhibitors on the vascular endothelium, including vildagliptin. However, the involved mechanisms are not yet clear. In this study, Sprague-Dawley rats were randomly divided into the following four groups: control, diabetic, diabetic + low-dose vildagliptin (10 mg/kg/d), and diabetic + high-dose vildagliptin (20 mg/kg/d). The diabetic model was created by feeding a high-fat diet for four weeks and injection of streptozotocin. Then, vildagliptin groups were given oral vildagliptin for twelve weeks, and the control and diabetic groups were given the same volume of saline. The metabolic parameters, endothelial function, and whole genome expression in the aorta were examined. After 12 weeks of treatment, vildagliptin groups showed significantly reduced blood glucose, blood total cholesterol, and attenuated endothelial dysfunction. Notably, vildagliptin may inhibit angiopoietin-like 3* (Angptl3)* and betaine-homocysteine S-methyltransferase* (Bhmt)* expression and activated paraoxonase-1* (Pon1)* in the aorta of diabetic rats. These findings may demonstrate the vasoprotective pathway of vildagliptin* in vivo*.

## 1. Introduction

The incidence and prevalence of diabetes mellitus are dramatically increasing worldwide [1]. Epidemiological research shows that diabetic patients have a higher risk of cardiovascular diseases [2]. The main cause of morbidity and mortality in diabetic patients is cardiovascular diseases.

Glucagon-like peptide-1 (GLP-1) is produced in gut L-cells. It contains 30 amino acids. The main physiological function of GLP-1 is stimulation of insulin secretion from pancreatic *β* cells when glucose is orally taken up in the human body. GLP-1 can also inhibit glucose production and appetite, activate adipose and muscle glucose uptake and storage, and thus moderate insulin sensitivity. However, GLP-1 is quickly hydrolyzed by dipeptidyl peptidase-4 (DPP-4).

DPP-4 inhibitors are a new class of GLP-1 based antidiabetic drugs. As one type of DPP-4 inhibitors, vildagliptin controls blood glucose by inhibiting the enzymatic activity of DPP-4. DPP-4 is also found on endothelial cells in the cardiovascular system, and increasing research has focused on the benefit of DPP-4 inhibitors on cardiovascular function. Sitagliptin (one type of DPP-4 inhibitor) has been proven to significantly attenuate heart failure-related left ventricular (LV) end-diastolic pressure, systolic performance, and chamber stiffness in an ablation-induced cardiac dysfunction rat model [3]. In a clinical trial, sitagliptin enhanced global and regional LV function in type 2 diabetes mellitus (T2DM) patients with coronary artery [4]. Vildagliptin exerts cardioprotective effects in obesity-based insulin resistance [5, 6], myocardial infarction (MI) [7], and ischemia-reperfusion (I/R) injury rat models [8]. However, the exact mechanism of the beneficial effect of vildagliptin on the aorta in diabetic rats remains to be elucidated.

In this study, we hypothesized that vildagliptin improved aorta function through multiple pathways. We employed a whole genomic expression array and bioinformatics method to explore the pathway involved in aorta vascular function moderation in diabetic rats.

## 2. Materials and Methods

### 2.1. Animal Treatments and Diets

Five-week-old male Sprague-Dawley rats were obtained from the Institute of Laboratory Animal Science, Chinese Academy of Medical Sciences, and Peking Union Medical College (Beijing, China, SCXK-2014-0013). The animal protocol was approved by the Animal Care Committee of the Peking Union Medical Hospital Animal Ethics Committee (Project XHDW-2015-0051, 15 Feb 2015), and all efforts were made to minimize suffering. All the rats were fed in a light/dark cycle (12 hours : 12 hours) environment and were free to drink water. Three days after arrival, rats were randomly divided into four groups (*n* = 6 per group): normal control group, diabetic group, low-dose vildagliptin (vil-low), and high-dose vildagliptin (vil-high). The normal control group was fed a standard rodent diet (kcal%: 10% fat, 20% protein, and 70% carbohydrate; 3.85 kcal/gm). Other groups were fed a high-fat diet (kcal%: 45% fat, 20% protein, and 35% carbohydrate; 4.73 kcal/gm, Research Diet, New Brunswick, NJ, USA). After 4 weeks, diabetic, low-dose vildagliptin, and high-dose vildagliptin groups were given a single injection of streptozotocin (STZ, 30 mg/kg body weight, i.p., Sigma-Aldrich, St. Louis, MO, USA). Fasting blood glucose > 11.1 mmol/L was the standard for the diabetic model. Then, vil-low and vil-high groups were treated with 10 mg or 20 mg vildagliptin (Novartis Pharma AG, Basel, Switzerland)/kg of body weight by daily gavage for 12 weeks. Normal control and diabetic groups were given normal saline. After 12 weeks of treatment, the rats were anesthetized using ketamine (100 mg/kg i.p., Pharmacia and Upjohn Ltd., Crawley, UK), followed by withdrawal of food overnight. Blood samples were obtained from the abdominal aorta. Then, the rats were sacrificed by decapitation. The thoracic aorta was quickly removed. Some aortas were placed in Krebs solution (120 mmol/L of NaCl, 4.7 mmol/L of KCl, 1.18 mmol/L of KH_2_PO_4_, 2.25 mmol/L of CaCl_2_, 24.5 mmol/L of NaHCO_3_, 1.2 mmol/L of MgSO_4_·7H_2_O, 11.1 mmol/L of glucose, and 0.03 mmol/L of EDTA) and aerated with 95% O_2_ and 5% CO_2_. Other aortas were frozen in liquid nitrogen and stored at −80°C for a gene microarray experiment.

### 2.2. Body Weight and Fasting Blood Glucose Measurements

The rats were weighed every 4 weeks. Fasting blood glucose levels were measured by Bayer Contour TS glucometer (Hamburg, Germany).

### 2.3. Oral Glucose Tolerance Test (OGTT)

An OGTT was performed after 12 weeks of treatment. Blood glucose levels were measured at 30, 60, and 120 min after an oral administration of 20% glucose at a dose of 2 g/kg. The area under the curve (AUC) was calculated by the linear trapezoid method [9].

### 2.4. Serum Insulin and Lipid Panel Measurements

Serum fasting insulin was analyzed using an ELISA kit (Millipore, Billerica, MA, USA). The homeostasis model assessment of insulin resistance (HOMA-IR) was calculated by the following formula: FBG (mmol/L) × fasting insulin (*μ*IU/mL)/22.5. Serum total cholesterol (TC), triglyceride (TG), high-density lipoprotein (HDL), and low-density lipoprotein (LDL) were measured using an enzyme end-point kit (Roche Diagnostics GmbH, Mannheim, Germany).

### 2.5. Isometric Contractile Tension Assay

Isometric contractile tension was determined as follows and described previously [10]. The aortic ring segments (3 mm width) were incubated with Krebs solution (120 mmol/L of NaCl, 4.7 mmol/L of KCl, 1.18 mmol/L of KH_2_PO_4_, 2.25 mmol/L of CaCl_2_, 24.5 mmol/L of NaHCO_3_, 1.2 mmol/L of MgSO_4_·7H_2_O, 11.1 mmol/L of glucose, and 0.03 mmol/L of EDTA), aerated with 95% O_2_ and 5% CO_2_, and then preconstricted with phenylephrine (Phe, 10^−7^ mmol/L). Isometric contractile tension was measured by a BL-410 Biological function system (Chengdu Tai Meng Science and Technology Co., Ltd., Chengdu, China). Once a basal contraction was obtained, 10^−10^ to 10^−4^ mol/L of acetylcholine (Ach) was cumulatively added to the solution.

### 2.6. RNA Extraction, Amplification, Labeling, and Hybridization

To look for the differentially expressed genes under vildagliptin treatment in diabetic rats, we performed gene whole transcript-based array in vil-high group and diabetic group (*n* = 3 in each group). Total RNA was extracted from aortas using a mirVana™ RNA Isolation Kit (Ambion, San Paulo, SP, Brazil). The RNA was purified using an RNeasy Kit (Qiagen, Hilden, Germany), quantified by NanoDrop ND-2000 spectrophotometry (Nanodrop Tech, Rockland, Del, Wilmington, DE, USA), and qualified by agarose gel electrophoresis. Total RNA (100 ng) was used for cDNA synthesis. cRNA was synthesized followed by two-strand cDNA. Biotin-labeled cRNA was hybridized to an Affymetrix GeneChip Rat Gene 2.0 ST whole transcript-based array (Affymetrix Technologies, Santa Clara, CA, USA). After hybridization, the microarrays were washed, stained, and scanned with an Affymetrix Scanner 3000 7G (Santa Clara, CA, USA).

The microarray signals were analyzed using Expression Console software (version 1.4.1, Affymetrix, Santa Clara, CA, USA). The significance of the difference in genes was determined by one-way ANOVA. Differentially expressed genes were defined as genes with a fold change > 2.0 and *P* < 0.05. The microarray raw data were submitted to the Gene Expression Omnibus (GEO) repository (GSE102196).

### 2.7. Bioinformatics Analysis for Microarray

DAVID (Database for Annotation, Visualization and Integrated Discovery) software (https://david.abcc.ncifcrf.gov/) [11] was used to perform gene ontology (GO) and Kyoto Encyclopedia of Genes and Genomes (KEGG) pathway enrichment analyses. The Search Tool for the Retrieval of Interacting Genes (STRING) database (https://string-db.org/) [12] was used to analyze the interaction network for differentially expressed genes.

### 2.8. Validation of Differentially Expressed Genes by Quantitative PCR

The expression levels of three differentially expressed genes were measured by qPCR to validate the results of the microarray. All primers are listed in Table 1.* Actin* was used as an internal control.

### 2.9. Statistical Analyses

Data are presented as the means ± standard deviation (SD). One-way ANOVA analysis followed by Student's *t*-test was used to compare differences among groups. *P* < 0.05 was considered significant.

## 3. Results

### 3.1. Vildagliptin Moderated Blood Glucose and Insulin Resistance

**Figure 1:**
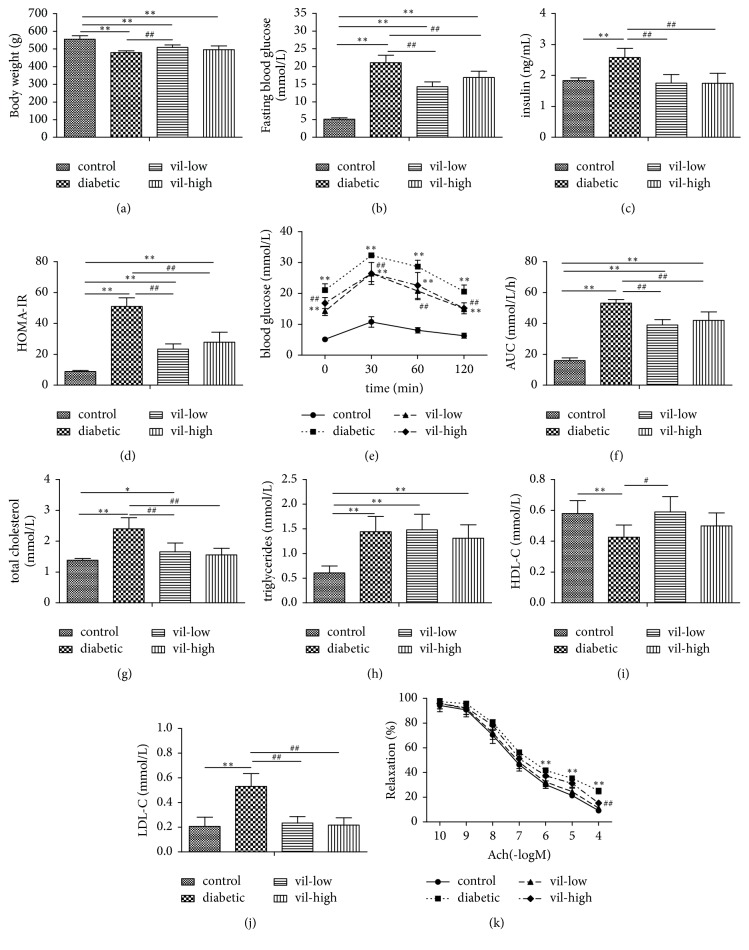
Effect of vildagliptin on metabolic parameters in rats. (a) Body weight, (b) fasting blood glucose, (c) insulin, (d) homeostasis model assessment (HOMA-IR), (e) blood glucose in oral glucose tolerance test (OGTT), (f) area under the curve (AUC) in OGTT, (g) total cholesterol, (h) triglyceride, (i) high-density lipoprotein cholesterol (HDL-C), (j) low-density lipoprotein cholesterol (LDL-C), and (k) relaxation responses to Ach in aortic rings. Values are mean ± SD (*n* = 6), ^*∗*^*P* < 0.05, ^*∗∗*^*P* < 0.01, versus control group; ^#^*P* < 0.05, ^##^*P* < 0.01 versus diabetic group. vil-low: low dose of vildagliptin; vil-high: high dose of vildagliptin.

After 12 weeks of treatment, vildagliptin significantly reduced fasting blood glucose AUC in OGTT (*P* < 0.05, Figures 1(b) and 1(f)). Additionally, fasting serum insulin and HOMA-IR index in the vildagliptin-treated group were lower than in the diabetic group (*P* < 0.05, Figures 1(c) and 1(d)).

### 3.2. Vildagliptin Decreased Blood Lipid Levels

Vildagliptin decreased TC and LDL-C levels in diabetic rats (*P* < 0.01, Figures 1(g) and 1(j)).

### 3.3. Vildagliptin Depressed Vasorelaxation Responses of Aortic Rings to Ach

As shown in Figure 1(k), endothelium-dependent vasodilation was impaired in diabetic rats compared with normal rats. Treatment with vildagliptin ameliorated this impairment (*P* < 0.01).

### 3.4. Identification of Differentially Expressed Genes

After filtering genes that had a cutoff of a 1.5-fold change or greater, we found that 150 genes were upregulated and 120 genes were downregulated in the vil-high group compared with the diabetic group (*P* < 0.05).

### 3.5. GO Term Enrichment Analysis of Differentially Expressed Genes

**Figure 2:**
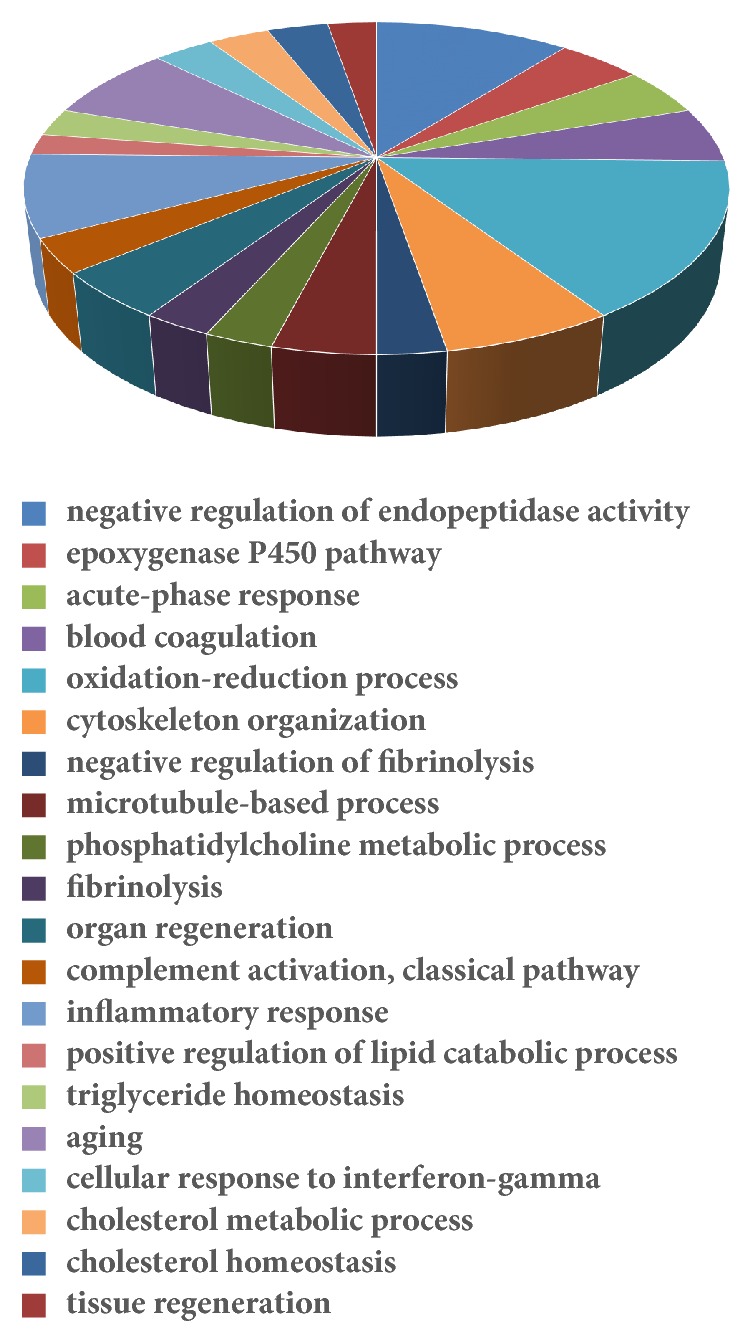
Enriched biological process for the differentially expressed genes between vil-high group and diabetic group.

In general, 91 GO terms were significantly enriched (*P* < 0.05, Table 2). The top 20 most significant biological process (BP) terms are shown in Figure 2 (*P* < 0.01). They were negative regulation of endopeptidase activity, epoxygenase P450 pathway, acute-phase response, blood coagulation, oxidation-reduction processes, cytoskeleton organization, negative regulation of fibrinolysis, microtubule-based processes, phosphatidylcholine metabolic processes, fibrinolysis, organ regeneration, complement activation classical pathway, inflammatory response, positive regulation of lipid catabolic process, triglyceride homeostasis, aging, cellular response to interferon gamma, cholesterol metabolic process, cholesterol homeostasis, and tissue regeneration.

### 3.6. Pathway Enrichment Analysis of Differentially Expressed Genes

In the pathway enrichment analysis, 12 pathway terms were significantly enriched (*P* < 0.05, Table 3). The top 10 most significant pathway terms were complement and coagulation cascades, retinol metabolism, steroid hormone biosynthesis, chemical carcinogenesis, linoleic acid metabolism, inflammatory mediator regulation of TRP channels, Gap junction, prion diseases, metabolic pathways, and arachidonic acid metabolism.

### 3.7. Gene Interaction Network

**Figure 3:**
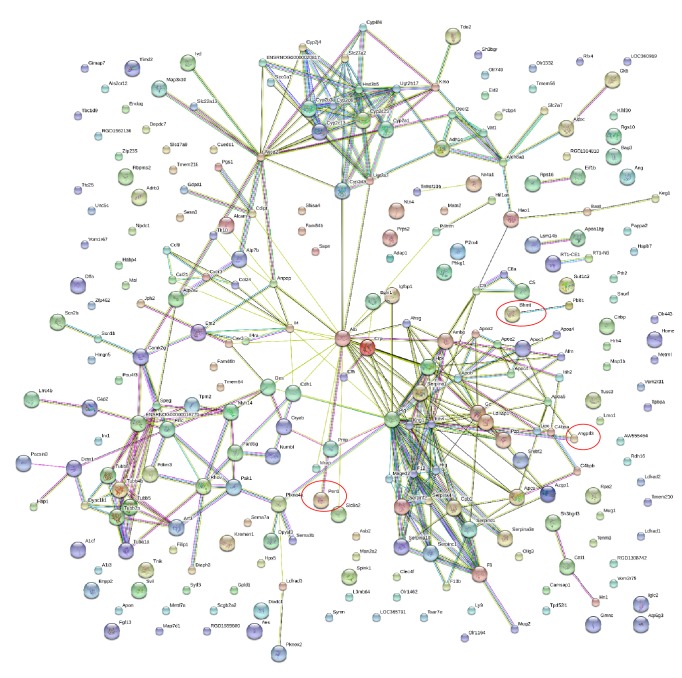
Interaction of differentially expressed genes between vil-high group and diabetic group from String software.* Angptl3*,* Bhmt*, and* Pon1* are circled in red.

Based on 270 differentially expressed genes, a gene interaction network was analyzed by the String software. We found that 257 nodes and 338 edges were constructed (Figure 3). Twenty-five genes had more than 5 edges. The top 10 genes by degree are listed in Table 4.

### 3.8. Confirmation with qPCR

**Figure 4:**
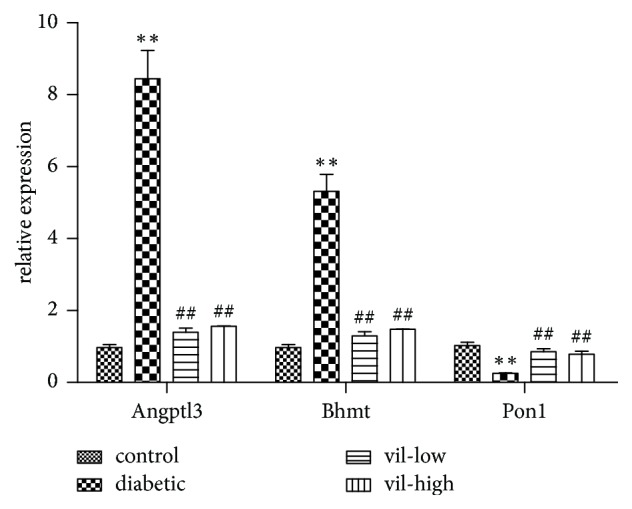
Confirmation of three representative differentially expressed genes by qPCR. Values are mean ± SD (*n* = 6).* Angptl3*: angiopoietin-like 3;* Bhmt*: betaine-homocysteine S-methyltransferase;* Pon1*: paraoxonase 1. ^*∗∗*^*P* < 0.01 versus control group; ^##^*P* < 0.01 versus diabetic group. vil-low: low dose of vildagliptin; vil-high: high dose of vildagliptin.

From the gene expression array results, we found that* Pon1* and* Bhmt* were in “metabolic pathway” (KEGG ID: rno01100), and* Angptl3* and* Pon1* were in several GO terms such as “positive regulation of lipid catabolic process” (GO: 0050996), “triglyceride homeostasis” (GO: 0070328), “cholesterol metabolic process” (GO: 0008203), “cholesterol homeostasis” (GO: 0042632), “response to hypoxia” (GO: 0001666), and “acylglycerol homeostasis” (GO: 0055090). So, we focused on these three genes in further study. As shown in Figure 4, the relative mRNA levels of angiopoietin-like 3* (Angptl3)* and betaine-homocysteine S-methyltransferase* (Bhmt)* in diabetic rats were significantly higher than those of normal rats (*P* < 0.01), whereas the expression of these two genes was significantly reduced in vildagliptin-treated groups compared with those in diabetic rats (*P* < 0.01). Conversely, paraoxonase 1* (Pon1)* decreased in the diabetic group. However, Pon1 increased in vildagliptin-treated rats (*P* < 0.01). These results were consistent with the microarray results.

## 4. Discussion

In our study, we found that vildagliptin reduced blood glucose, TC, and LDL-C. In a clinical trial, sitagliptin-combined metformin add-on therapy led to greater improvement in HbA1c than the metformin monotherapy group after 18 weeks of treatment. Moreover, sitagliptin combined with metformin significantly reduced TG, TC, and LDL-C and increased HDL-C compared with the metformin group [13].

We used aortic rings to test the Ach-induced endothelium-dependent vasodilation. In diabetic rats, Ach-induced endothelium-dependent vasodilation was impaired. Vildagliptin augmented endothelial function. Other DDP-4 inhibitors also had similar vasoprotective effects. Saxagliptin treatment for 8 weeks increased aortic nitric oxide (NO) release by 22% and reduced mean arterial pressure in spontaneously hypertensive rats [14]. Moreover, sitagliptin treatment for 2 weeks protected endothelial function and reduced systolic blood pressure in spontaneously hypertensive rats through GLP-1 signaling [15].

In our research, the expression of* Angptl3* was downregulated in vildagliptin-treated rats.* Angptl3* is a key regulator, which can inhibit lipoprotein lipase (LPL) activity [16, 17]. The enzyme LPL hydrolyzes TG to free fatty acids (FFA). ANGPTL3 overexpression mice had high plasma TG levels [18]. Patients with a loss-of-function mutation of* Angptl3* are characterized by low plasma TC, TG, HDL-C, and LDL-C [19, 20]. In 2008, Kathiresan et al. first identified an SNP site near ANGPTL3 that was associated with plasma TG levels in a genome-wide association study [21]. In obesity and T2D subjects, the ANGPTL3 level was higher than that in healthy subjects [22]. Moreover, the level of ANGPTL3 was increased in the livers of insulin-deficient and insulin-resistant diabetic mice [23]. In mice and monkeys, using ANGPTL3-specific antibodies led to reduced plasma TG [24–26]. The therapeutic targets of the inhibition activity of ANGPTL require further research to treat dyslipidemia [16, 17].

We also found that the expression of* Bhmt* was reduced in the vildagliptin-treated group. Methionine (Met) was produced from methylate homocysteine (Hcy) by BHMT enzymes with betaine. Thus, BHMT can reduce Hcy levels and increase Met levels. Met can then be converted to S-adenosylmethionine (SAM).* In vivo*, SAM is a main methyl donor to regulate methionine metabolism in many reactions. An increase in BHMT activity and SAM levels was observed in the livers of both type 1 diabetic rats [27] and the type 2 diabetic model [28, 29]. Insulin treatment in the rat hepatoma cell line and STZ-induced diabetic rats can inhibit the excess expression of BHMT and SAM [28, 30].

Moreover, our results showed that Pon1 was upregulated in the vildagliptin-treated group. Pon1 is an enzyme that has antioxidant functions. In serum, Pon1 is located in HDL. Pon1 protects LDL from oxidation and hydrolyzed oxidized LDL [31–34]. HDL levels are negatively associated with the risk of developing coronary artery disease (CAD), but high levels of oxidized LDL (oxLDL) in the aorta lead to cholesterol accumulation, foam cell formation, and atherosclerotic lesions [31, 35]. Low Pon1 expression accelerates aortic lesion development in mice [36]. In humans, serum PON level is reduced in patients with a history of myocardial infarction [32] and diabetic patients [37]. PON1 transgenic mice had decreased oxidative stress and atherosclerotic lesions [38, 39], whereas PON1 knockout mice had increased serum oxidative stress and were more susceptible to high-fat-diet-induced atherosclerosis [34, 40].

## 5. Conclusion

**Figure 5:**
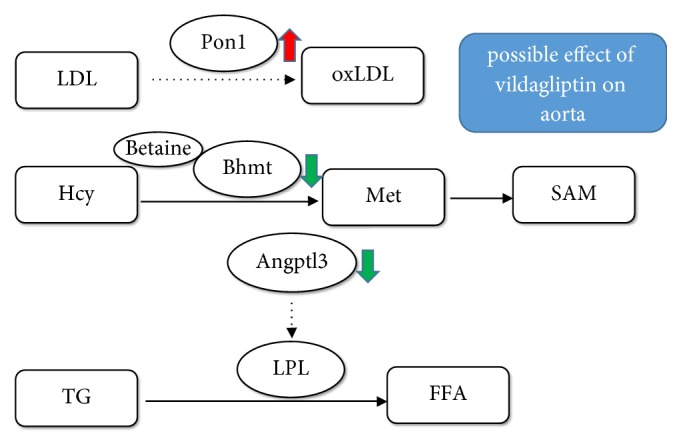
*The possible mechanism of attenuation of vildagliptin on endothelial dysfunction*. Vildagliptin attenuates endothelial dysfunction in diabetic rats through activating* Pon1* expression in aorta to inhibit oxLDL production; inhibiting Bhmt expression to reduce Met and SAM production; inhibiting* Angptl3* expression to activate LPL activity and thereby reduce plasma TG level. LDL: low-density lipoprotein; Pon1: paraoxonase 1; oxLDL: oxidized low-density lipoprotein; Hcy: homocysteine; Bhmt: betaine-homocysteine S-methyltransferase; Met: methionine, SAM: S-adenosylmethionine; TG: triglyceride.

In conclusion, this study has confirmed that vildagliptin significantly attenuates endothelial dysfunction in diabetic rats. Importantly, our study indicates that vildagliptin may activate* Pon1* expression and inhibit the expression of* Bhmt* and* Angptl3* in the aorta. This study may increase the understanding of the pathways that contribute to which DPP-4 inhibitor attenuates endothelial dysfunction. More studies are needed to investigate whether or not vildagliptin treatment directly alters* Agptl3*,* Bhmt*, and* Pon1* expression in the aorta of diabetic rats, independent of blood glucose (Figure 5).

## Figures and Tables

**Table 1:** Oligonucleotide sequences for qPCR analysis.

Gene symbol	GenBank ID	Forward primer	Reverse Primer	Product size
*Angptl3*	NM_001025065	AAAGGGTTTTGGGAGGCTTGA	CCCAAAAGCGCTATGGTCTC	117
*Bhmt*	NM_030850	GATGCTTGGGGAGTGACGAA	TGTGGCTACTGTGCGGATTT	119
*Pon1*	NM_032077	AAGCTGGCTACACCCACATC	CAACATTCGTTGGTGAGCGG	103

*Angptl3*: angiopoietin-like 3; *Bhmt*: betaine-homocysteine S-methyltransferase; *Pon1*: paraoxonase 1.

**Table 2:** The enriched GO terms with differentially expressed genes (*P* < 0.05).

Term ID	Term name	Count	*P*-value	Fold Enrichment	catalogue	Genes
GO:0010951	negative regulation of endopeptidase activity	16	7.22 × 10^−10^	8.425	Biology Process	KNG1, MUG2, MUG1, SERPINA10, PZP, C5, AHSG, AMBP, SERPINA3N, SERPINF2, SERPINA4, SERPINC1, ITIH4, HRG, ITIH2, SERPIND1
GO:0019373	epoxygenase P450 pathway	7	5.88 × 10^−7^	21.821	Biology Process	CYP2C6V1, CYP2B3, CYP2J4, CYP2C23, CYP2C13, CYP2A1, CYP2A2
GO:0006953	acute-phase response	7	7.97 × 10^−6^	14.356	Biology Process	KNG1, MUG1, CRP, ITIH4, SAA4, SERPINA1, AHSG
GO:0007596	blood coagulation	8	2.08 × 10^−5^	9.446	Biology Process	F13B, F12, SERPINA10, C9, F9, SERPIND1, CPB2, PLG
GO:0055114	oxidation-reduction process	23	3.29 × 10^−5^	2.753	Biology Process	ALDH8A1, CYP2B3, CYP2J4, HSD3B5, DECR2, RGD1304810, CYP2D3, EGLN1, KMO, P4HTM, VAT1, IYD, CYP2C6V1, TDO2, CYP3A23/3A1, HIF1AN, CYP4F4, CYP2C23, CYP2C13, SH3BGRL3, RDH16, CYP2A1, CYP2A2
GO:0007010	cytoskeleton organization	10	3.95 × 10^−5^	6.089	Biology Process	CCL24, TNIK, CAP2, TUBB2A, SVIL, CAMSAP1, STRIP2, TUBB6, TUBA1A, TUBB4B
GO:0051918	negative regulation of fibrinolysis	4	6.93 × 10^−5^	44.533	Biology Process	SERPINF2, HRG, CPB2, PLG
GO:0007017	microtubule-based process	6	1.68 × 10^−4^	11.405	Biology Process	TUBB2A, TUBB5, TUBB6, TUBA1A, DCTN1, TUBB4B
GO:0046470	phosphatidylcholine metabolic process	4	6.75 × 10^−4^	22.267	Biology Process	APOA5, PON1, GPLD1, LIPC
GO:0042730	fibrinolysis	4	8.35 × 10^−4^	20.782	Biology Process	F12, HRG, CPB2, PLG
GO:0031100	organ regeneration	7	0.00110	5.995	Biology Process	APOA2, BAAT, ADH1, ALDOC, APOA5, PRPS2, AHSG
GO:0006958	complement activation, classical pathway	5	0.00123	10.532	Biology Process	C9, C5, CRP, C4BPB, C4BPA
GO:0006954	inflammatory response	12	0.00158	3.149	Biology Process	CCL24, KNG1, TLR10, SERPINA3N, TNFRSF10B, MUG1, CCL21, C5, HRH4, CCL9, SERPINA1, CXCR3
GO:0050996	positive regulation of lipid catabolic process	3	0.00236	38.967	Biology Process	APOA2, APOA5, ANGPTL3
GO:0070328	triglyceride homeostasis	4	0.00431	11.990	Biology Process	APOC4, APOA5, LIPC, ANGPTL3
GO:0007568	aging	11	0.00745	2.721	Biology Process	ADRB3, CYP3A23/3A1, CRYAB, ENDOG, ALDOC, CRP, SPINK1, IGFBP1, ATP5G3, SREBF2, HTR2A
GO:0071346	cellular response to interferon-gamma	5	0.00766	6.388	Biology Process	CCL24, SERPINA3N, CCL21, CCL9, CFH
GO:0008203	cholesterol metabolic process	5	0.00811	6.285	Biology Process	APOA2, PON1, LIPC, ANGPTL3, SREBF2
GO:0042632	cholesterol homeostasis	5	0.00906	6.089	Biology Process	CAV3, APOA2, APOA5, LIPC, ANGPTL3
GO:0042246	tissue regeneration	4	0.00996	8.907	Biology Process	SERPINA10, APOA5, IGFBP1, PLG
GO:0046330	positive regulation of JNK cascade	5	0.0112	5.730	Biology Process	DIXDC1, CCL21, SERPINF2, MAP3K10, TPD52L1
GO:0050921	positive regulation of chemotaxis	3	0.0134	16.700	Biology Process	CCL21, C5, CXCR3
GO:0006956	complement activation	3	0.0153	15.587	Biology Process	C8A, C5, CFH
GO:2000649	regulation of sodium ion transmembrane transporter activity	3	0.0153	15.587	Biology Process	CAV3, SCN1B, SCN2B
GO:0042573	retinoic acid metabolic process	3	0.0173	14.613	Biology Process	ALDH8A1, ADH1, RDH16
GO:0009395	phospholipid catabolic process	3	0.0217	12.989	Biology Process	APOA2, ENPP2, ANGPTL3
GO:0001666	response to hypoxia	9	0.0230	2.598	Biology Process	CRYAB, ANG, CAMK2G, ALDOC, CRP, SERPINA1, EGLN1, PAK1, LIPC
GO:0055117	regulation of cardiac muscle contraction	3	0.0241	12.305	Biology Process	CAV3, P2RX4, CALM3
GO:0055090	acylglycerol homeostasis	2	0.0254	77.933	Biology Process	APOA5, ANGPTL3
GO:0034370	triglyceride-rich lipoprotein particle remodeling	2	0.0254	77.933	Biology Process	APOA2, APOA5
GO:0031116	positive regulation of microtubule polymerization	3	0.0265	11.690	Biology Process	CAV3, MAP1B, DCTN1
GO:0032956	regulation of actin cytoskeleton organization	4	0.0287	5.995	Biology Process	DIXDC1, HRG, PAK1, SH3BGRL3
GO:0048247	lymphocyte chemotaxis	3	0.0317	10.627	Biology Process	CCL24, CCL21, CCL9
GO:0070098	chemokine-mediated signaling pathway	4	0.0363	5.469	Biology Process	CCL24, CCL21, CCL9, CXCR3
GO:0045959	negative regulation of complement activation, classical pathway	2	0.0378	51.956	Biology Process	C4BPB, C4BPA
GO:0002542	Factor XII activation	2	0.0378	51.956	Biology Process	KNG1, F12
GO:0009725	response to hormone	5	0.0381	3.936	Biology Process	ANG, APOA5, LIPC, ANGPTL3, SREBF2
GO:0042311	vasodilation	3	0.0402	9.352	Biology Process	KNG1, P2RX4, ALB
GO:0006631	fatty acid metabolic process	4	0.0413	5.196	Biology Process	BAAT, DECR2, LIPC, ANGPTL3
GO:0007399	nervous system development	7	0.0428	2.741	Biology Process	PLXNA4, SCN2B, CAMK2G, KREMEN1, MAP1B, DPYSL3, NUMBL
GO:0048675	axon extension	3	0.0431	8.992	Biology Process	SEMA7A, MAP1B, POU4F3
GO:0072659	protein localization to plasma membrane	4	0.0485	4.871	Biology Process	CAV3, TNIK, FGF13, CDH1
GO:0006935	chemotaxis	4	0.0485	4.871	Biology Process	CCL24, ENPP2, C5, CXCR3
GO:0006810	transport	6	0.0489	3.017	Biology Process	P2RX4, DYNC1LI1, AFM, LOC360919, ALB, LOC500473
GO:0072562	blood microparticle	22	1.84 × 10^−18^	14.631	Cellular Components	KNG1, GC, C9, MUG1, APCS, C4BPA, PLG, AHSG, C8A, AMBP, APOA2, AFM, SERPINF2, ALB, HPX, APOA5, PON1, CFH, SERPINC1, ITIH4, HRG, ITIH2
GO:0005615	extracellular space	49	2.19 × 10^−11^	2.922	Cellular Components	MUG2, MUG1, PZP, CRP, GPLD1, SPINK1, ANPEP, AZGP1, APOA2, ANG, SEMA7A, SERPINA4, APOA5, CFH, SERPINA1, SEMA3B, KNG1, F12, APCS, LOC360919, F9, C8A, AMBP, SERPINA3N, SERPINF2, SCGB2A2, GC, SERPINA10, ENPP2, C5, CCL9, AHSG, CCL24, ALB, CCL21, SERPINC1, ANGPTL3, C4BPB, DPYSL3, C4BPA, PLG, AFM, HPX, PON1, METRNL, SERPIND1, IGFBP1, LIPC, CPB2
GO:0034364	high-density lipoprotein particle	7	4.31 × 10^−8^	32.313	Cellular Components	APOA2, APOC4, APOA5, PON1, GPLD1, SAA4, LIPC
GO:0070062	extracellular exosome	66	6.06 × 10^−8^	1.957	Cellular Components	ALDH8A1, CYP2J4, MUG1, TUBB2A, CRP, GPLD1, ANPEP, CKB, AZGP1, APOA2, DES, PACSIN3, ANG, SERPINA4, ITIH4, TUBB5, CFH, TUBB6, ITIH2, SEMA3B, SERPINA1, TUBA1A, KNG1, F12, TNIK, APCS, CRYAB, F9, METTL7A, VAT1, AMBP, C8A, RPS16, SERPINF2, BHMT, PRNP, SLC27A2, PRPS2, GC, C9, SERPINA10, ALDOC, C5, CDH1, KMO, AHSG, ALCAM, ALB, SERPINC1, DOPEY2, HRG, TUBB4B, COTL1, PLG, P2RX4, AFM, HPX, ARF3, PON1, CALM3, PAPPA2, METRNL, SERPIND1, SH3BGRL3, MYH14, CPB2
GO:0031090	organelle membrane	11	2.10 × 10^−7^	9.591	Cellular Components	CYP2C6V1, CYP2B3, UGT2B37, UGT2B17, CYP3A23/3A1, CYP4F4, CYP2C23, CYP2D3, CYP2C13, CYP2A1, CYP2A2
GO:0030018	Z disc	9	1.24 × 10^−4^	6.037	Cellular Components	CAV3, JPH2, DES, CRYAB, BAG3, PDLIM3, PAK1, HOMER1, FLNC
GO:0014704	intercalated disc	6	4.60 × 10^−4^	9.232	Cellular Components	CAV3, SCN1B, DES, ATP2A2, FGF13, PAK1
GO:0030426	growth cone	9	6.13 × 10^−4^	4.772	Cellular Components	ANG, CRP, MAP1B, CALM3, DPYSL3, FGF13, MYH14, PAK1, HAP1
GO:0005874	microtubule	11	8.97 × 10^−4^	3.658	Cellular Components	DYNC1LI1, TUBB2A, CAMSAP1, MAP1B, TUBB5, TUBB6, FGF13, TUBA1A, DCTN1, TUBB4B, GLYATL2
GO:0043034	costamere	4	0.00196	15.695	Cellular Components	SVIL, SYNM, HOMER1, FLNC
GO:0005576	extracellular region	19	0.00456	2.088	Cellular Components	APCS, C9, CRP, NTN4, GPLD1, SAA4, C4BPB, FGF13, PTH2, C4BPA, PLG, LOC500473, CCL24, C8A, SERPINA3N, ALB, APOA5, HRG, LIPC
GO:0005789	endoplasmic reticulum membrane	16	0.00504	2.258	Cellular Components	CYP2B3, CDIPT, HSD3B5, CYP2D3, SREBF2, CYP2C6V1, UGT2B37, UGT2B17, CYP3A23/3A1, ATP2A2, CYP4F4, CYP2C23, CYP2C13, CYP2A1, CYP2A2, SLC27A2
GO:0030424	axon	12	0.00657	2.609	Cellular Components	GC, ALCAM, P2RX4, CRYAB, ALDOC, MAP1B, FGF13, CDH1, MYH14, PAK1, HOMER1, HTR2A
GO:0034361	very-low-density lipoprotein particle	3	0.0192	13.848	Cellular Components	APOA2, APOC4, APOA5
GO:0043231	intracellular membrane-bounded organelle	16	0.0302	1.825	Cellular Components	KNG1, CYP2B3, CAV3, CYP2J4, HSD3B5, GPLD1, PLG, SREBF2, AMBP, UGT2B17, CYP3A23/3A1, PON1, CYP2C23, UGT2A3, CYP2A1, SLC27A2
GO:0005856	cytoskeleton	8	0.0345	2.605	Cellular Components	TNIK, DES, FILIP1, MAP1B, FLNC, COTL1, TPM2, HAP1
GO:0044216	other organism cell	2	0.0376	52.316	Cellular Components	C4BPB, C4BPA
GO:0033017	sarcoplasmic reticulum membrane	3	0.0457	8.719	Cellular Components	JPH2, ATP2A2, CAMK2G
GO:0004867	serine-type endopeptidase inhibitor activity	15	6.34 × 10^−11^	11.167	Molecular Function	MUG2, PZP, MUG1, SERPINA10, SPINK1, AMBP, SERPINA3N, SERPINF2, SERPINA4, SERPINC1, ITIH4, HRG, ITIH2, SERPINA1, SERPIND1
GO:0070330	aromatase activity	9	2.65 × 10^−8^	18.039	Molecular Function	CYP2C6V1, CYP2B3, CYP3A23/3A1, CYP4F4, CYP2C23, CYP2D3, CYP2C13, CYP2A1, CYP2A2
GO:0008392	arachidonic acid epoxygenase activity	8	1.97 × 10^−7^	18.393	Molecular Function	CYP2C6V1, CYP2B3, CYP2J4, CYP2C23, CYP2D3, CYP2C13, CYP2A1, CYP2A2
GO:0008395	steroid hydroxylase activity	8	5.35 × 10^−7^	16.035	Molecular Function	CYP2C6V1, CYP2B3, CYP2J4, CYP2C23, CYP2D3, CYP2C13, CYP2A1, CYP2A2
GO:0020037	heme binding	13	1.46 × 10^−6^	6.122	Molecular Function	CYP2B3, CYP2J4, CYP2D3, AMBP, CYP2C6V1, TDO2, CYP3A23/3A1, CYP4F4, CYP2C23, HRG, CYP2C13, CYP2A1, CYP2A2
GO:0008092	cytoskeletal protein binding	8	1.07 × 10^−5^	10.423	Molecular Function	DES, PACSIN3, CRYAB, ALDOC, PDLIM3, CDH1, FLNC, ABCG2
GO:0005506	iron ion binding	13	1.11 × 10^−5^	5.031	Molecular Function	CYP2B3, CYP2J4, CYP2D3, EGLN1, P4HTM, CYP2C6V1, CYP3A23/3A1, HIF1AN, CYP4F4, CYP2C23, CYP2C13, CYP2A1, CYP2A2
GO:0004866	endopeptidase inhibitor activity	5	5.40 × 10^−4^	13.028	Molecular Function	MUG2, MUG1, C5, SERPINA1, AHSG
GO:0017080	sodium channel regulator activity	5	6.95 × 10^−4^	12.214	Molecular Function	CAV3, SCN1B, SCN2B, GPLD1, FGF13
GO:0016712	oxidoreductase activity, acting on paired donors, with incorporation or reduction of molecular oxygen, reduced flavin or flavoprotein as one donor, and incorporation of one atom of oxygen	5	8.78 × 10^−4^	11.495	Molecular Function	CYP2B3, CYP2J4, CYP3A23/3A1, CYP2D3, CYP2A1
GO:0005200	structural constituent of cytoskeleton	6	0.00223	6.514	Molecular Function	TUBB2A, TUBB5, TUBB6, SYNM, TUBA1A, TUBB4B
GO:0016706	oxidoreductase activity, acting on paired donors, with incorporation or reduction of molecular oxygen, 2-oxoglutarate as one donor, and incorporation of one atom each of oxygen into both donors	4	0.00262	14.213	Molecular Function	HIF1AN, RGD1304810, EGLN1, P4HTM
GO:0008201	heparin binding	8	0.00268	4.283	Molecular Function	SERPINA10, ANG, APOA5, SERPINC1, CFH, HRG, SERPIND1, LIPC
GO:0016705	oxidoreductase activity, acting on paired donors, with incorporation or reduction of molecular oxygen	5	0.00297	8.316	Molecular Function	CYP2C6V1, CYP2B3, CYP2C23, EGLN1, CYP2C13
GO:0005102	receptor binding	12	0.00666	2.598	Molecular Function	KNG1, HAO1, P2RX4, BAAT, ANG, LRRC4B, DECR2, HRG, HOMER1, SLC27A2, HAP1, PLG
GO:0004497	monooxygenase activity	5	0.00944	6.013	Molecular Function	CYP2C6V1, CYP2B3, CYP2C23, CYP2D3, CYP2C13
GO:0004622	lysophospholipase activity	3	0.01328	16.751	Molecular Function	ENPP2, LIPC, GDPD1
GO:0042803	protein homodimerization activity	19	0.01474	1.845	Molecular Function	RBPMS2, CRYAB, GIMAP7, CAMK2G, CRP, NR4A1, TPD52L1, ABCG2, AMBP, ADRB3, APOA2, HIF1AN, ANG, SERPINF2, ADH1, TENM3, PON1, RDH16, PRPS2
GO:0005543	phospholipid binding	5	0.0198	4.825	Molecular Function	APOA2, APOA5, MAP1B, PON1, SYTL3
GO:0044325	ion channel binding	6	0.0233	3.693	Molecular Function	CAV3, CALM3, FGF13, PRNP, HOMER1, HAP1
GO:0048020	CCR chemokine receptor binding	3	0.0239	12.342	Molecular Function	CCL24, CCL21, CCL9
GO:0043395	heparan sulfate proteoglycan binding	3	0.0289	11.167	Molecular Function	CFH, HRG, LIPC
GO:0030215	semaphorin receptor binding	3	0.0289	11.167	Molecular Function	SEMA7A, SEMA3B, SH3BGRL3
GO:0005509	calcium ion binding	16	0.0330	1.797	Molecular Function	MATN2, F12, APCS, CALR4, TBC1D9, ENPP2, CRP, DECR2, F9, CDH1, P4HTM, ATP2A2, PON1, ITIH4, CALM3, SYTL3
GO:0008307	structural constituent of muscle	3	0.0371	9.771	Molecular Function	PDLIM3, SYNM, TPM2
GO:0003779	actin binding	8	0.0406	2.511	Molecular Function	GC, DIXDC1, CAP2, ANG, DIAPH3, MAP1B, COTL1, TPM2
GO:0015020	glucuronosyltransferase activity	3	0.0460	8.685	Molecular Function	UGT2B37, UGT2B17, UGT2A3
GO:0005507	copper ion binding	4	0.0463	4.963	Molecular Function	P2RX4, ANG, PRNP, ATP7B
GO:0005515	protein binding	29	0.0484	1.427	Molecular Function	CAV3, GC, SCN1B, ALDOC, CRP, CDH1, RHOV, CKB, A1CF, TUBB5, SERPINC1, SERPINA1, PAK1, TUBA1A, HAP1, TUBB4B, SCN2B, CRYAB, MAP1B, HRK, NR4A1, HOMER1, NUMBL, P2RX4, ATP2A2, CALM3, SLC27A2, PRPS2, HTR2A

**Table 3:** The enriched Kegg pathway with differentially expressed genes (*P* < 0.05).

Pathway ID	Pathway name	Count	*P*-value	Fold Enrichment	Genes
rno04610	Complement and coagulation cascades	16	7.92 × 10^−14^	14.904	KNG1, F12, C9, C5, F9, C4BPB, C4BPA, PLG, F13B, C8A, SERPINF2, CFH, SERPINC1, SERPINA1, SERPIND1, CPB2
rno00830	Retinol metabolism	12	3.00 × 10^−8^	9.697	CYP2C6V1, CYP2B3, UGT2B37, UGT2B17, CYP3A23/3A1, ADH1, CYP2C23, CYP2C13, UGT2A3, CYP2A1, RDH16, CYP2A2
rno00140	Steroid hormone biosynthesis	10	2.63 × 10^−6^	8.280	CYP2C6V1, CYP2B3, UGT2B37, UGT2B17, CYP3A23/3A1, HSD3B5, CYP2C23, CYP2D3, CYP2C13, UGT2A3
rno05204	Chemical carcinogenesis	9	5.47 × 10^−5^	6.633	CYP2C6V1, CYP2B3, UGT2B37, UGT2B17, CYP3A23/3A1, ADH1, CYP2C23, CYP2C13, UGT2A3
rno00591	Linoleic acid metabolism	5	3.01 × 10^−3^	8.179	CYP2C6V1, CYP2J4, CYP3A23/3A1, CYP2C23, CYP2C13
rno04750	Inflammatory mediator regulation of TRP channels	7	6.98 × 10^−3^	4.082	CYP2C6V1, CYP2J4, CAMK2G, CYP2C23, CALM3, CYP2C13, HTR2A
rno04540	Gap junction	6	9.57 × 10^−3^	4.573	TUBB2A, TUBB5, TUBB6, TUBA1A, TUBB4B, HTR2A
rno05020	Prion diseases	4	1.14 × 10^−2^	8.384	C8A, C9, C5, PRNP
rno01100	Metabolic pathways	30	1.17 × 10^−2^	1.556	CYP2B3, CYP2J4, PGS1, CDIPT, TUSC3, ALDOC, HSD3B5, KMO, ANPEP, ATP5G3, CKB, TDO2, ADH1, CYP2C13, UGT2A3, CYP2C6V1, MAN2A2, HAO1, UGT2B37, UGT2B17, BAAT, CYP3A23/3A1, BHMT, PON1, CYP2C23, LIPC, RDH16, CYP2A1, CYP2A2, PRPS2
rno00590	Arachidonic acid metabolism	5	3.16 × 10^−2^	4.140	CYP2C6V1, CYP2B3, CYP2J4, CYP2C23, CYP2C13
rno04726	Serotonergic synapse	6	3.63 × 10^−2^	3.245	CYP2C6V1, CYP2J4, CYP2C23, CYP2D3, CYP2C13, HTR2A
rno04360	Axon guidance	6	4.08 × 10^−2^	3.144	PLXNA4, SEMA7A, NTN4, SEMA3B, PAK1, UNC5C

**Table 4:** The top 10 genes from gene interaction analysis.

Gene Accession	Gene Symbol	gene name	degree
NM_019369	Itih4	inter-alpha-trypsin inhibitor heavy chain family, member 4	17
NM_053491	Plg	plasminogen	16
NM_001014006	F12	coagulation factor XII (Hageman factor)	12
NM_001012027	Serpinc1	serpin peptidase inhibitor, clade C (antithrombin), member 1	11
NM_138514	Cyp2c13	cytochrome P450, family 2, subfamily c, polypeptide 13	9
NM_019287	Apob	apolipoprotein B	8
ENSRNOT00000074103	Cyp2c6	cytochrome P450, family 2, subfamily C, polypeptide 6, variant 1	8
NM_053318	Hpx	hemopexin	8
NM_022519	Serpina1	clade A (alpha-1 antiproteinase, antitrypsin), member 1	8
NM_001108802	Speg	SPEG complex locus	7

## Data Availability

The data used to support the findings of this study are included within the article.
